# Prioritization of therapeutic targets for cancers using integrative multi-omics analysis

**DOI:** 10.1186/s40246-024-00571-2

**Published:** 2024-04-24

**Authors:** Xin Jin, Yunyun Mei, Puyu Yang, Runze Huang, Haifeng Zhang, Yibin Wu, Miao Wang, Xigan He, Ziting Jiang, Weiping Zhu, Lu Wang

**Affiliations:** 1 Department of Hepatic Surgery, Fudan University Shanghai Cancer Center, Shanghai Medical College, Fudan University, Shanghai, 200032 People’s Republic of China; 2 Department of Oncology, Shanghai Medical College, Fudan University, Shanghai, 200032 People’s Republic of China; 3 Department of Neurosurgery, Fudan University Shanghai Cancer Center, Shanghai Medical College, Fudan University, Shanghai, 200032 People’s Republic of China; 4 Department of Epidemiology, School of Public Health, Medical College of Soochow University, Suzhou, People’s Republic of China; 5 Department of Endoscopy, Fudan University Shanghai Cancer Center, Shanghai Medical College, Fudan University, Shanghai, 200032 People’s Republic of China

**Keywords:** Cancers, Genome-wide association studies, Therapeutic targets, Multi-omics, NRF2 pathway

## Abstract

**Background:**

The integration of transcriptomic, proteomic, druggable genetic and metabolomic association studies facilitated a comprehensive investigation of molecular features and shared pathways for cancers’ development and progression.

**Methods:**

Comprehensive approaches consisting of transcriptome-wide association studies (TWAS), proteome-wide association studies (PWAS), summary-data-based Mendelian randomization (SMR) and MR were performed to identify genes significantly associated with cancers. The results identified in above analyzes were subsequently involved in phenotype scanning and enrichment analyzes to explore the possible health effects and shared pathways. Additionally, we also conducted MR analysis   to investigate metabolic pathways related to cancers.

**Results:**

Totally 24 genes (18 transcriptomic, 1 proteomic and 5 druggable genetic) showed significant associations with cancers risk. All genes identified in multiple methods were mainly enriched in nuclear factor erythroid 2-related factor 2 (NRF2) pathway. Additionally, biosynthesis of ubiquinol and urate were found to play an important role in gastrointestinal tumors.

**Conclusions:**

A set of putatively causal genes and pathways relevant to cancers were identified in this study, shedding light on the shared biological processes for tumorigenesis and providing compelling genetic evidence to prioritize anti-cancer drugs development.

**Supplementary Information:**

The online version contains supplementary material available at 10.1186/s40246-024-00571-2.

## Background

As a prevalent chronic disease, cancer arises from the uncontrolled proliferation of abnormal cells, posing a significant threat to human health. Statistically, there were nearly 19.3 million new cancer cases and almost 10.0 million cancer-related deaths in 2020 [1]. Due to the complex and multifaceted mechanism underlying tumorigenesis, the treatment of cancers remains a challenge. Meanwhile, numerous ongoing clinical trials are assessing the efficacy of new drugs as cancer therapeutics. Despite efforts, approximately 90% of drugs that progress into clinical trials ultimately fail, primarily due to insufficient efficacy or safety concerns. This contributes to an astonishing average cost of $1.3 billion to complete the development and commercialization of a new drug [2–4].

Increasing evidence suggests that drug targets with genetic support usually exhibit a higher success rate in clinical trials and ultimately deliver more effective treatments to patients in need [5–7]. As a powerful research approach, Genome-wide association studies (GWAS) offer comprehensive genomic data, enabling researchers to investigate molecules and pathways involved in the development of diseases.

Some analysis methods based on GWAS data like transcriptome-wide association studies (TWAS), proteome-wide association studies (PWAS), summary-data-based Mendelian randomization (SMR) and colocalization have been widely used to inform potential drug targets, which present unprecedented opportunities to develop novel drugs for many complex diseases [7, 8]. In fact, some drugs developed based on genetic research, such as PCSK9 [9], CCR5 [10] and ACE2 [11], have already yielded successful outcomes, advancing the treatment of related diseases.

In this study, we sought to ascertain novel therapeutic targets for cancers with the multi-omics GWAS data. An integrative analysis was adopted to investigate candidate genes for cancers at the transcriptomic and proteomic level. Utilizing eQTL and pQTL data, we performed TWAS/PWAS analysis separately to identify casual gene transcripts and proteins for cancers primarily. Then, SMR/MR, Bayesian colocalization and differential expression analysis were leveraged to further confirm above results. What’s more, druggable genomic and metabolic data were also included to enrich our research findings through MR analysis. Our study comprehensively prioritized candidate genes for cancers based on multi-omics genetic data, contributing to a better understanding of the potential mechanisms and addressing challenges in the lengthy and costly process of novel drugs development.

## Methods

### Study design and ethics

**Fig. 1:**
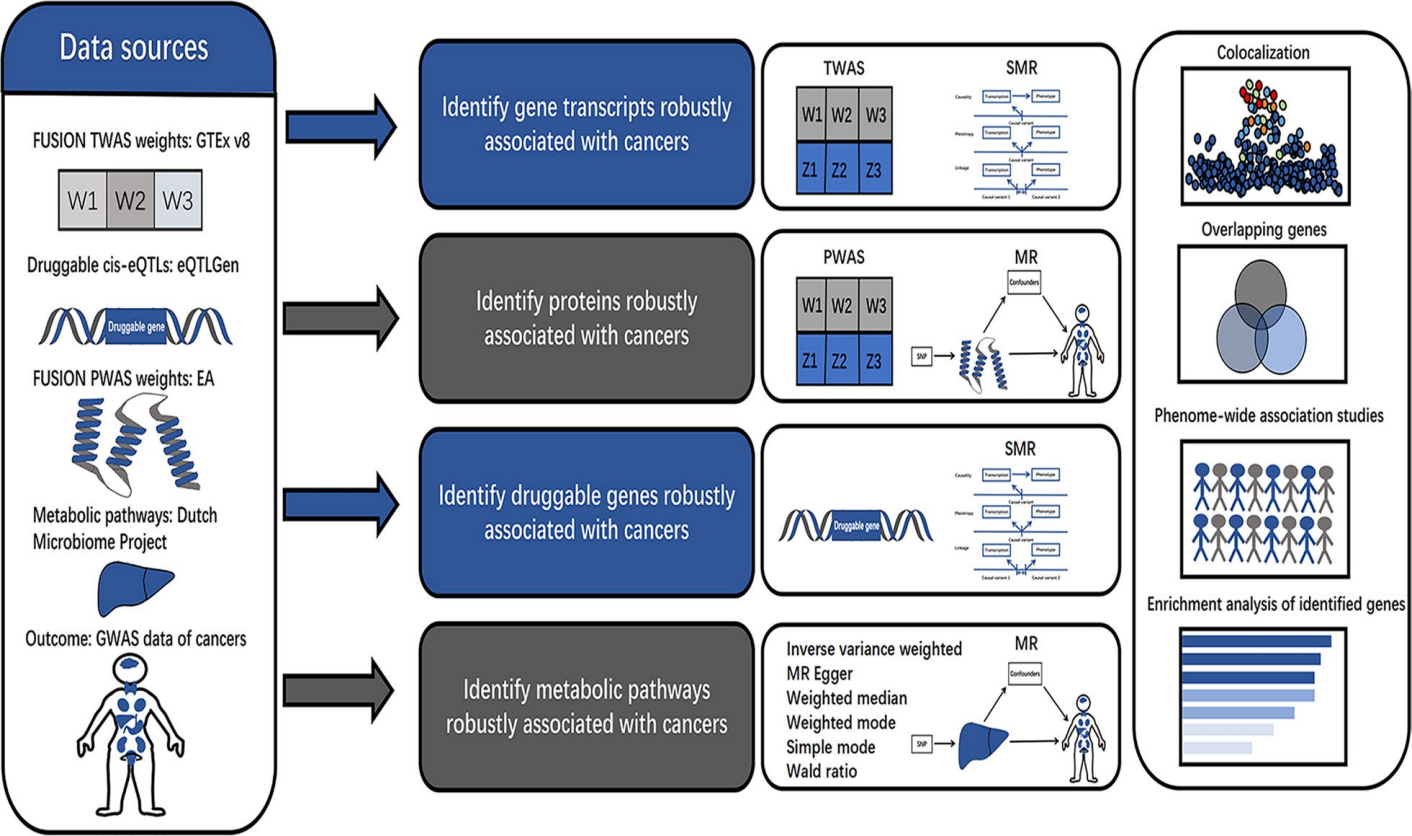
Study design and flow diagrams. The transcriptomic, proteomic, druggable genetic and metabolomic association with cancers were recognized through comprehensive methods. 18 gene transcripts (TWAS-significant, SMR-significant and PP.H4 > 0.8), 1 protein-coding genes (PWAS-significant, MR-significant and PP.H4 > 0.8) and 5 druggable genes (SMR-significant and PP.H4 > 0.8) were included in phenotype scanning and enrichment analysis. Additionally, we conducted two-samples MR analyzes to identify 2 metabolic pathways significantly associated with cancers. *TWAS* transcriptome-wide association studies, *PWAS* proteome-wide association studies, *MR* Mendelian randomization, *SMR* summary-data-based Mendelian randomization, *PP.H4* posterior probability that two traits are associated with a single causal variant, *eQTL* expression quantitative trait loci, *GTEx v8* genotype-tissue expression project version 8, *EA* European American

The overall study design and methods are presented in Fig. 1. The included studies have undergone ethical review and obtained approval from review committees.

### Outcome sources

GWAS summary statistics for cancers were extracted from the FinnGen R9 release. FinnGen study is a large-scale study that combines genome information with digital healthcare data of 500,000 Finnish individuals, aiming to identify new therapeutic targets and diagnostics for treating numerous diseases through genetic research and improve human health [12]. Totally 16 types of cancers were involved as outcomes in our study and details were presented in Additional file 1: Table S1.

### TWAS (transcriptomics)

TWAS integrates GWAS and gene expression data to identify specific genes or genetic variants that contribute to the observed trait or disease [13]. Functional Summary-based Imputation (FUSION), a widely used tool for TWAS analysis, establishes  precomputed predictive models from multiple studies to facilitate testing comprehensive associations throughout the transcriptome (http://gusevlab.org/projects/fusion/) [13]. In this study, genes expression weights generated from Genotype-Tissue Expression Project version 8 (GTEx v8) serves as a reference framework to illustrate intricate associations between Single nucleotide polymorphisms (SNPs) and genes expression, encompassing whole blood and corresponding organ tissue panels. 1000 Genomes European samples (https://data.broadinstitute.org/alkesgroup/FUSION/LDREF.tar.bz2) were utilized to estimate the Linkage disequilibrium (LD) between the prediction model and the SNP at each locus of GWAS. Each gene experienced the permutation test for 2000 times by *Z*-test. Specifically, we set a Bonferroni-corrected threshold at *P* < (0.05/number of features) to mitigate the increased likelihood of false positive results that arises when conducting multiple statistical tests simultaneously (Additional file 1: Table S2).

### SMR (transcriptomics)

As an extension and development of the concept of MR, SMR analysis tests whether the effect of SNPs on cancers is mediated through gene expression, prioritizing genes responsible for tumorigenesis. SMR analysis was conducted with the default settings through the command line interface (https://yanglab.westlake.edu.cn/software/smr/#Overview) [14]. The heterogeneity in dependent instruments (HEIDI) test was further employed to determine whether the identified association between genes expression and cancers was attributable to linkage. The two-sided *P* < 0.01 in HEIDI test demonstrates that the correlation is most likely due to linkage [14].

### Bayesian colocalization (transcriptomics)

To test whether the genetic associations with both identified genes and cancers shared the single causal variants, we employed colocalization analyzes for TWAS-significant and SMR-significant results [15]. All SNPs within 1 Mb range upstream and downstream of each leading SNP were included in this analysis and the posterior probability of H4 (PP.H4) > 0.8 indicated that identified genes colocalized strongly with cancers. We selected TWAS-significant, SMR-significant and PP.H4 > 0.8 genes as high confidence genes (HCG).

### PWAS (proteomics)

The same FUSION workflow was applied for PWAS with the default settings and parameters. In this study, we analyzed 1348 circulating proteins from 7213 European American (EA) in the Atherosclerosis Risk in Communities study (http://nilanjanchatterjeelab.org/pwas/), combining the corresponding European ancestry sample LD reference [16].

### MR (proteomics)

Two-sample MRs were performed to capture the causal associations between the plasma levels of proteins and cancers risk refer to the study by Ferkingstad et al. [17], containing 4907 different blood proteins measured in 35,559 Icelanders. The proteins with quantitative trait loci (pQTLs)^−^ were involved in the MR analysis. Wald ratio was performed when a single pQTL was available for a given protein, and inverse variance weighted (IVW) was applied when multiple genetic instruments were accessible. Same as TWAS analysis, we also performed colocalization to further screen the above results. PWAS-significant, MR-significant and PP.H4 > 0.8 genes were defined as HCG.

### Druggable SMR (genomics)

The druggable genome was defined as a collection of genes that encoded targetable proteins, including compounds in clinical trials, approved medications and small compounds validated in preclinical experiments [18]. Focusing on this subset of genes, we aimed to identify further potential repurposing opportunities to inform trials of cancer patients. In this study, the cis-eQTLs extracted from the eQTLGen Consortium were utilized to generate genetic instruments for druggable genome. Within 1 Mb on either side of the encoded gene, common (minor allele frequency [MAF] > 1%) cis-eQTLs that demonstrated a significant association (*P* < 5.0 × 10^−8^) with the expression of druggable genes were selected. Moreover, HEIDI tests and colocalization were applicated in this section. We described the genes meeting the criteria of Druggable SMR-significant and PP.H4 > 0.8 as HCG.

### Metabolome-wide MR (metabolomics)

In order to elucidate metabolic mechanisms underlying tumorigenesis, we conducted metabolome-wide MR analyzes for 205 metabolic pathways on outcomes (Additional file 1: Table S18). Genetic data of metabolic pathway was obtained from a genome-wide association study called Dutch Microbiome Project, aiming to demonstrate the interaction between host genetics and microbial composition and function [19]. To begin with, we employed a rigorous criterion (*P* < 1 × 10^−5^) to ensure a comprehensive outcome. All instrumental variables (IVs) subsequently underwent Linkage disequilibrium (LD) clumping (*r*2 = 0.001; distance = 10,000 kb) to mitigate the potential influence of SNP correlations. SNPs located outside the major histocompatibility complex (MHC) region (chr6, 26–34 Mb) were excluded. The *F*-statistic of the selected SNPs should exceed a threshold of 10.

### Differential expression analysis

We further performed a differential expression analysis to verify the role therapeutic targets plays in specific tumor. The transcriptome RNA-seq and clinical data for above genes were extracted from the Cancer Genome Atlas (TCGA) database (https://portal.gdc.cancer.gov/) and GTEx (https://gtexportal.org/home/).

### Phenotype scanning

Aiming to investigate possible health effects of HCG, we conducted phenotype scanning in MR analyzes with publicly available electronic health record data corresponding to 1293 health-related endpoints (number of cases > 1000) in FinnGen Release 5. The Bonferroni correction is a common method designed to control the increased risk of a type I error when making multiple statistical tests. According to Bonferroni correction, the results with a *P* values less than 0.05/number of health-related endpoints were considered to be significant. Hence, we reported all gene-trait associations significant under a Bonferroni-corrected threshold of *P* < 3.87 × 10^−5^ (0.05/1293).

### Enrichment analysis

Enrichment analysis were conducted to explore the shared mechanism contributing to cancers in the Metascape database (http://www.metascape.org/) [20], limiting the species to “Homo sapiens”, and setting the cut-off *P* value as 0.01 and min overlap as three.

## Results

### Transcriptomic association studies (TWAS, SMR, colocalization)

This study employed TWAS, SMR, and colocalization to impute robust gene expression signatures associated with cancers. TWAS analysis revealed significant associations between the expression of 151 genes and cancers in whole blood and specific organ tissues. Meanwhile,  SMR anaysis identified 52 genes whose expression in whole blood and specific organs tissue were associated with cancers. To test whether these genes and cancers shared the single causal variants, the TWAS-significant, SMR-significant results were subsequently refined through colocalization analyzes. A total of 18 genes’ expression colocalized strongly with the cancers (PP.H4 > 0.80), which was recognized as HCG. HCG and detailed results were presented in Table 1 and Additional file 1: Tables S4–S8.

**Table 1:** High confidence genes associated with cancers (TWAS significant, SMR significant, and PP.H4 > 0.8)

Type	Gene	TWAS			SMR					Coloc
		Model	TWAS.Z	TWAS.P	topSNP	eQTL beta	SMR beta	OR (95% CI)	*P* val	PP.H4
Kidney cancer (whole blood)	IRF5	susie	− 4.65	0.00000328	rs3778754	0.4	− 0.40	0.67 (0.57–0.78)	0.000000412	0.99
Lung (tissue)	AMT	susie	− 4.6	0.00000413	rs113741748	− 0.45	− 0.24	0.79 (0.71–0.87)	6.14E−06	0.87
Breast Mammary Tissue (tissue)	MRPS30	enet	7.61	2.84E−14	rs7716571	0.23	0.47	1.59 (1.32–1.93)	1.68E−06	0.99
Breast Mammary Tissue (tissue)	RCCD1	top1	− 5.18	0.000000221	rs113343095	0.55	− 0.17	0.85 (0.79–0.91)	3.01E−06	0.98
Malignant melanoma of skin (whole blood)	HERC2P9	lasso	− 6.66	2.82E−11	rs2003233	− 0.4	− 0.78	0.46 (0.35–0.6)	1.63E−08	0.87
Skin Not Sun Exposed (tissue)	CLPTM1L	enet	− 8.11	5.12E−16	rs31490	− 0.14	− 1.62	0.2 (0.11–0.35)	1.45E−08	0.97
Skin Not Sun Exposed (tissue)	SPATA2L	lasso	6.92	4.63E−12	rs34404057	− 0.15	1.51	4.54 (2.66–7.75)	2.84E−08	0.92
Skin Not Sun Exposed (tissue)	GAS8	enet	7.06	1.63E−12	rs12930631	− 0.29	0.73	2.07 (1.51–2.84)	6.22E−06	0.82
Skin Sun Exposed (tissue)	CLPTM1L	susie	− 8.76	2.00E−18	rs401681	− 0.12	− 1.81	0.16 (0.08–0.34)	1.44E−06	0.98
Prostate cancer (whole blood)	EEFSEC	top1	− 7.57	3.85E−14	rs4857836	0.13	− 0.91	0.4 (0.29–0.56)	9.07E−08	0.94
Prostate (tissue)	IRX4	top1	− 8.49	1.99E−17	rs530543670	− 0.53	− 0.26	0.77 (0.72–0.83)	2.05E−12	0.99
Prostate (tissue)	TPCN2	enet	7.79	6.47E−15	rs72932523	0.47	0.26	1.3 (1.16–1.46)	6.08E−06	1
Prostate (tissue)	PPP1R14A	top1	9.99	1.65E−23	rs12610267	− 0.2	0.70	2.02 (1.56–2.61)	7.96E−08	0.99
Gastric cancer	THBS3	enet	5.08	0.000000371	rs760077	− 0.41	0.56	1.75 (1.42–2.14)	0.000000113	0.99
Gastric (tissue)	PSCA	top1	6.47	9.71E−11	rs2976388	0.7	0.37	1.44 (1.28–1.62)	1.05E−09	0.96
Gastric (tissue)	LY6K	susie	6.88	6.10E−12	rs2976388	0.43	0.60	1.82 (1.4–2.38)	1.04E−05	0.97
Gastric (tissue)	THEM6	enet	5.23	0.000000169	rs2717600	0.26	0.93	2.54 (1.75–3.68)	9.01E−07	0.89
Thyroid (tissue)	SMAD3	susie	− 6.89	5.58E−12	rs56375023	− 0.48	− 0.50	0.61 (0.51–0.72)	1.23E−08	0.98

High confidence genes associated with cancers reached significant thresholds of reached TWAS analysis, SMR analysis and colocalization analysis. The expression weights of TWAS analysis and cis-eQTLs from SMR analysis were generated from whole blood and corresponding organ tissue from the GTEx v8 release. The genes significant in both TWAS and SMR analyzes were then assessed in colocalization analyzes to further test robustness. TWAS transcriptome-wide association study, SMR Summary-data-based Mendelian randomization, eQTL expression quantitative trait Loci, GTEx v8 genotype-tissue expression project version 8, OR odds ratio, PP.H4 posterior probability that two traits are associated with a single causal variant, SNP single nucleotide polymorphism

### Proteomic association studies (PWAS, MR, colocalization)

Same as above analysis, a set of methods containing PWAS, MR, colocalization were employed to establish reliable protein-coding gene expression signatures associated with cancers. Totally 17 protein-coding genes were identified through PWAS analysis in this study. Moreover, there were 6 protein-coding genes showing significant results in MR analysis. Those that are significant in both two methods were selected to perform a colocalization. Finally, only one protein-coding genes, PDCD6IP, colocalized strongly with the cancers (PP.H4 > 0.80) and was identified as HCG. Additional file 1: Table S9–S11 summarized the detailed information generated from the above analysis.

### Druggable genome-wide genetic association studies (SMR, colocalization)

Totally 2511 druggable genes’ cis-eQTLs were selected for drug-target SMR analyzes with a Bonferroni-corrected threshold of *P* < 1.99 × 10^−5^ (*P* < 0.05/2511). Totally 10 druggable genes was identified to have association signals with cancers through SMR analysis but only half of them had high support for colocalization with cancers, demonstrated by Table 2 and Additional file 1: Table S13 and S14. Decreased expression of “APOBEC3A” (OR 0.85; 95% CI 0.80–0.89; *P* = 1.51 × 10^−9^) and “NEK10” gene (OR 0.21; 95% CI 0.11–0.40; *P* = 1.69 × 10^−6^) were observed to be associated with the lower risk of   breast cancer. However, decreased expression of “GPX1” (OR 2.35; 95% CI 1.63–3.39; *P* = 5.42 × 10^−6^) and “THBS3” (OR 1.50; 95% CI 1.30–1.73; *P* = 3.30 × 10^−8^) were found to be associated with the higher risk of lung cancer and gastric cancer. Significant associations between the expression of “CASP9” (OR 0.66; 95% CI 0.54–0.79; *P* = 1.32 × 10^−5^) with kidney cancer were also mentioned.

**Table 2:** Druggable genes significantly associated with cancers (SMR significant and coloc > 0.8)

Type	Gene	SMR					Coloc
		topSNP	eQTL beta	SMR beta	OR (95% CI)	*P* val	PP.H4
Breast cancer	APOBEC3A	rs12628403	− 0.74	− 0.17	0.85 (0.80–0.89)	1.51E−09	1.00
Breast cancer	NEK10	rs62255653	− 0.06	− 1.55	0.21 (0.11–0.40)	1.69E−06	0.87
Lung cancer	GPX1	rs11130203	− 0.11	0.85	2.35 (1.63–3.39)	5.42E−06	0.92
Kidney cancer	CASP9	rs4233533	0.33	− 0.42	0.66 (0.54–0.79)	1.32E−05	0.94
Gastric cancer	THBS3	rs760077	− 0.56	0.41	1.50 (1.30–1.73)	3.30E−08	0.99

We perform SMR analysis and colocalization analysis to identify druggable genes significantly associated with cancers. The cis-eQTLs within 1 Mb on either side of the encoded gene extracted from the eQTLGen Consortium were used in SMR analysis. HEIDI tests and Bayesian colocalization were further conducted to assess the impact of pleiotropy. SMR analysis and colocalization analysis, eQTL expression quantitative trait loci, OR odds ratio, PP.H4 posterior probability that two traits are associated with a single causal variant, SNP single nucleotide polymorphism

### Metabolomics association studies (MR)

As shown in Table 3, the result showed a significant association between urate biosynthesis and biliary cancer (OR 2.77, 95% CI 1.80–4.26, *P* = 3.30 × 10^−6^) at a Bonferroni-corrected threshold of *P* < 2.44 × 10^−4^ (*P* < 0.05/205). Notably, the ubiquinol biosynthesis was found to be significantly associated with multiple gastrointestinal tumors like pancreatic cancer (OR 1.49, 95% CI 1.31–1.70; *P* = 1.26 × 10^−9^), liver cancer (OR 0.51, 95% CI 0.41–0.65; *P* = 2.10 × 10^−8^) and biliary cancer (OR 0.72, 95% CI 0.62–0.84, *P* = 2.37 × 10^−5^). The information of instrumental variable and results were illustrated Additional file 1: Tables S19 and S20.

**Table 3:** The results of metabolome-wide MR on cancers

Type	Path	Method	NSNP	OR (95% CI)	*P* val
Intrahepatic ducts, biliary tract and gallbladder cancer	PWY.5695..urate.biosynthesis.inosine.5..phosphate.degradation	Inverse variance weighted	8	2.77 (1.80–4.26)	3.30E−06
Intrahepatic ducts, biliary tract and gallbladder cancer	UBISYN.PWY..superpathway.of.ubiquinol.8.biosynthesis..prokaryotic	Inverse variance weighted	15	0.72 (0.62–0.84)	2.37E−05
Liver cancer	UBISYN.PWY..superpathway.of.ubiquinol.8.biosynthesis..prokaryotic	Inverse variance weighted	15	0.51 (0.41–0.65)	2.10E−08
Pancreatic cancer	UBISYN.PWY..superpathway.of.ubiquinol.8.biosynthesis..prokaryotic	Inverse variance weighted	15	1.49 (1.31–1.70)	1.26E−09

*NSNP* number of single nucleotide polymorphism, *OR* odds ratio

### Differential expression analysis

The results suggested that investigated genes have significant differential expression in specific tumors compared with normal tissues except for “APOBEC3A” and “NEK10”. However, the analysis conducted solely based on TCGA has yielded a reverse result, with these two genes marked as strongly significant genes to induce the breast cancer. The box plots of differential expression were illustrated in Additional file 2: Figs. S4–S7.

### Phenotype scanning

**Fig. 2:**
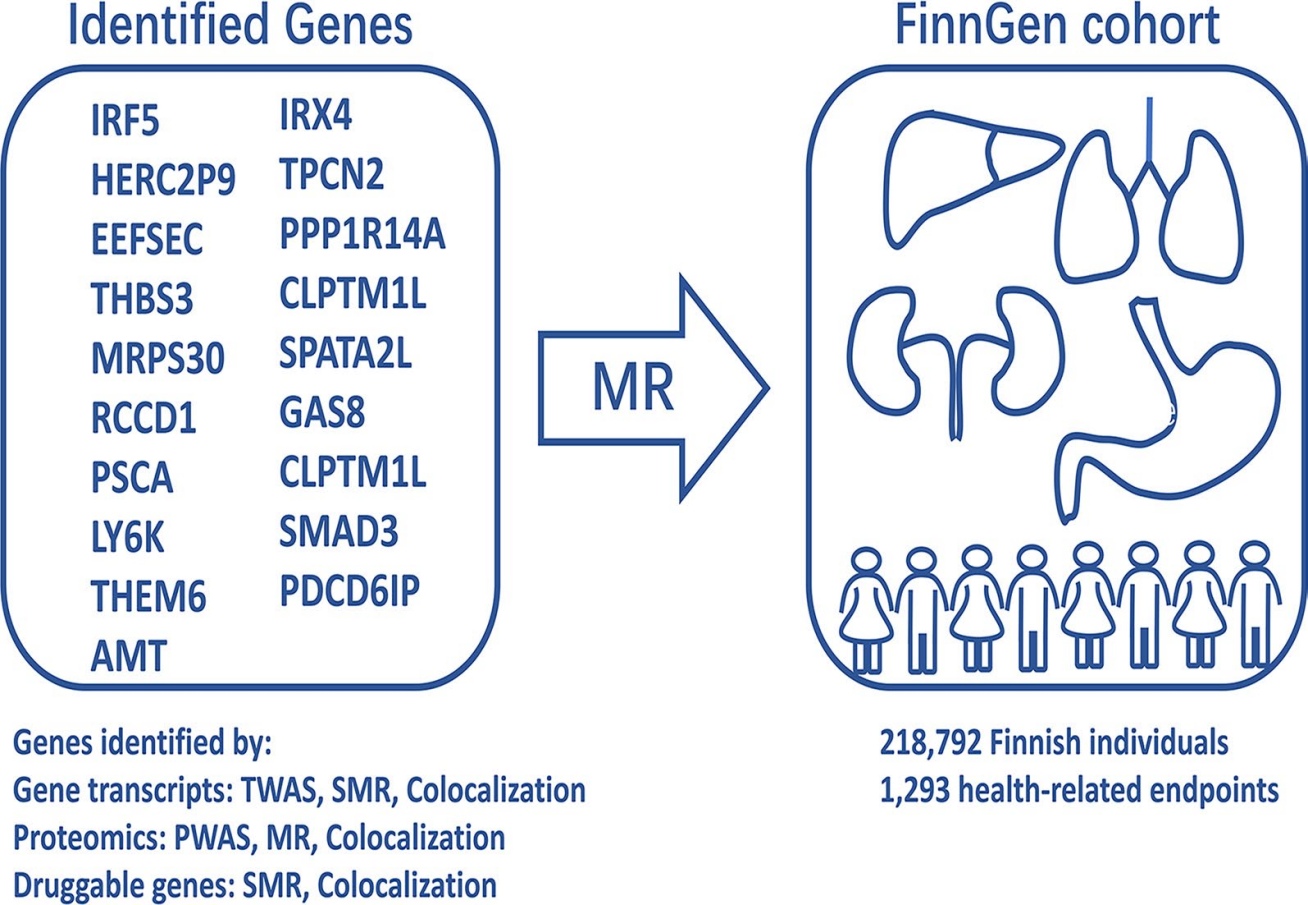
Phenotype scanning of the genes identified in the above analysis. Phenotype scanning was peroformed to investigate possible health effects of the genes identified in the previous analysis, using the publicly available electronic health record data corresponding to 1293 health-related endpoints (number of cases > 1000) in FinnGen Release 5

To investigate the potential impacts of pharmacologically targeting on our genetically identified genes, we carried out MR analyzes with a Bonferroni-corrected threshold of *P* < 3.87 × 10^−5^ in FinnGen Release 5 databse. SNPs located near genes, EEFSEC and TPCN2, were found to be associated with increased risk of asthma, autoimmune and inflammatory diseases. Conversely, SNPs located near gene, GPX, were associated with decreased risk of gastrointestinal-related diseases, such as inflammatory bowel disease and ulcerative colitis (Additional file 1: Table S15). Figure 2 summaries the workflow of phenotype scanning on candidate genes.

### Enrichment analysis

Functional enrichment analysis contributes to summarizing the common mechanisms underlying cancers development. Identified genes were mainly enriched in nuclear erythroid factor 2-related factor 2 (NRF2) pathway, regulating the cellular antioxidant response (Additional file 1: Tables S16 and S17, Additional file 2: Fig. S8).

## Discussion

In this study, we are committed to leveraging genetic data to picture the molecular characteristics and pathways associated with cancers. Through a variety of analytical methods, we jointly target 24 genes (18 transcriptomic, 1 proteomic and 5 druggable genome-wide genetic) and NRF2 pathway significantly associated with tumorigenesis. Furthermore, two metabolic pathways (PWY.5695..urate.biosynthesis.inosine.5..phosphate.degradation and UBISYN.PWY..superpathway.of.ubiquinol.8.biosynthesis..prokaryotic) were highlighted to be associated with the increased risk of gastrointestinal cancers.

In gene transcripts analysis, some results were confirmed by previous studies. As a glycosylphosphatidylinositol-anchored cell surface protein, PSCA is known to play a key role in intracellular signaling and tumor proliferation [21]. Indeed, the application of CAR NK cells targeting PSCA has exhibited extraordinary effectiveness in treating metastatic pancreatic cancer [22]. Nonetheless, there remains few studies regarding the association between gastric cancer and PSCA, warranting further investigation. Additionally, Iroquois-class homeodomain protein (IRX4) isoforms was identified to induce distinct functional programming, thereby contributing to suppressing the progression of prostate cancer [23, 24]. Meanwhile, a bioinformatic study indicated a significant correlation between the protein phosphatase 1 regulatory inhibitor subunit 14A (PPP1R14A) expression and the prognosis of patients of diverse tumor types across TCGA cohort, adding to the understanding of our results [25].

Among druggable genes identified in our study, APOBEC3A has received more attention. APOBEC3-associated mutations play an important role in the development of breast cancer and APOBEC3A was recently reported to be the main driver of these mutations [26–28]. Despite the conflict in the above analysis regarding the expression of APOBEC3A, we still encourage more explorations for APOBEC3A based on previous studies. Meanwhile, targeted therapy-induced APOBEC3A increases genomic instability and drives evolution of drug-tolerant persisters, suggesting that inhibition of APOBEC3A expression or activity may be an effective therapeutic strategies to reverse drug resistance [29]. What’s more, GPX1, ubiquitously expressing in many tissues, has been reported to have an aberrant expression in multiple cancers and be closely associated with oncogenesis and cancer progression [30]. However, there is some controversy regarding its impact on cancer susceptibility [31–35]. Its dichotomous roles as both a tumor suppressor and promoter in the specific cancer type should be noticed.

NRF2, a crucial regulator of the cellular antioxidant response, has been increasingly recognized as a driver of cancer progression, metastasis, and therapy resistance [21, 22, 36]. It is reported that NRF2 has played a direct role through upregulation of its target genes and an indirect role through redox modulation in tumorigenesis.  [23, 24]. Similarly, our results provide genetic evidence to further confirm an important role NRF2 pathway played in the tumor-related physiological. These promising discoveries indicated that NRF2 pathway warranted further investigation as a prognostic biomarker and a therapeutic target.

As one of the major hallmarks of malignancy, metabolic reprogramming plays a crucial role in tumor growth, progression and metastasis. To meet the enhanced requirements for biological processes essential for proliferation and survival, cancer cells undergo intrinsic modifications of the metabolic properties and preferences by regulating the flow of metabolic pathways [25, 37]. Notably, the biosynthesis of ubiquinol was highlighted as specific pathways for the risk of various gastrointestinal tumors. It’s reported that ubiquinol drives the oxidative tricarboxylic acid cycle and dihydroorotate dehydrogenase activity in mitochondrial electron transport chain, which is necessary for tumor growth [38–40]. However, the association between ubiquinol with gastrointestinal tumors has not been reported, requiring further studies.

While clinical trials are usually regarded as the gold standard for evaluating treatment efficacy and safety, it is important to recognize that bioinformatics analysis serves a different purpose and complements the findings from clinical trials. It allows for the exploration of large-scale genomic and molecular data, providing a comprehensive understanding of biological processes and disease mechanisms. Using computational tools and algorithms, bioinformatics can uncover complex relationships between genetic variations, gene expression patterns, and disease phenotypes, which can help researchers to identify relevant biomarkers and potential drug targets. Moreover, by aggregating and analyzing data from various studies, bioinformatics can provide a broader perspective and increase statistical power, which may not be feasible within the confines of a single clinical trial. However, it is important to acknowledge the limitations of bioinformatics analysis. The reliability of the results depends on the quality and accuracy of the input data, as well as the robustness of the analytical methods employed. Hence, the results generated from bioinformatics analysis may require a validation through experimental studies and clinical trials.

Some limitations need to be acknowledged. Firstly, due to the limited availability of multi-omics datasets, the reference data in our study predominantly consist of participants of European ancestry, demonstrating that the findings cannot be directly generalized to other ethnic groups. Secondly,  despite the exclusion of potential bias arising from linkage disequilibrium through colocalization analysis and HEIDI test, it is not possible to completely eliminated the impact of horizontal pleiotropy. Finally, it is noteworthy that the results generated from bioinformatics analysis may be considered less reliable compared to those derived from rigorous clinical trials. Therefore, additional clinical trials are needed to further assess the efficacy and safety of these findings.

In conclusion, our study successfully integrated transcriptomic, proteomic, druggable genetic and metabolomics association studies to explore molecular features and shared pathways underlying cancers’ incidence and progression, advancing the development of new drugs.

### Supplementary Information

**Additional file 1.** **Table S1.** The information of outcomes resources. **Table S2**. The information of exposures resources. **Table S3**. The information of TWAS-significant genes in whole blood. **Table S4**. Results of SMR for genes associated with cancers in whole blood. **Table S5**. Results of colocalization for the genes signficantly associated wtith cancers in TWAS and SMR analysis in whole blood. **Table S6**. The information of TWAS-significant genes in specific organ tissues. **Table S7**. Results of SMR for genes associated with cancers in specific organ tissues. **Table S8**. Results of colocalization for the genes signficantly associated wtith cancers in TWAS and SMR analysis in specific organ tissues. **Table S9**. The information of PWAS-significant genes in whole blood. **Table S10**. Results of MR for protein-code genes associated with cancers in whole blood. **Table S11**. Results of colocalization for protein-code genes signficantly associated wtith cancers in PWAS and MR analysis in whole blood. **Table S12**. The list of druggable genes for SMR.  **Table S13**. Results of SMR for druggable genes associated with cancers. **Table S14**. Results of colocalization for identified druggable genes wtith cancers in FinnGen cohort. **Table S15**. Results of phenotype scanning on candidate genes. **Table S16**. The information of enrichment analysis for candidate genes. **Table S17**. Results of enrichment analysis for candidate genes. **Table S18**. The information of metabolic pathways. **Table S19**. The information of IVs for Metabolome-wide Mendelian Randomization on cancers. **Table S20**. Results of Metabolome-Wide Mendelian Randomization on cancers.

**Additional file 2. Fig. S1.** Results of TWASs analysis on cancers in whole blood. **Fig. S2**. Results of TWASs analysis on cancers in specific organ tissue. **Fig. S3**. Results of PWASs analysis on cancers in whole blood. **Fig. S4**. Results of differential expression analysis on genes identified in transcriptomic association studies from TCGA and GTEx Database. **Fig. S5**. Results of differential expression analysis on genes identified in proteomic association studies from TCGA and GTEx Database. **Fig. S6**. Results of differential expression analysis on genes identified in druggable genomics association studies from TCGA and GTEx Database. **Fig. S7**. Results of differential expression analysis on genes identified in druggable genomics association studies from TCGA Database. **Fig. S8**. Results of enrichment analysis on the genes significantly associated with cancers.

## Data Availability

The datasets analyzed  in the current study are available in the following repository: GWAS data of cancers were extracted from the FinnGen R9: https://r9.finngen.fi/; The weights used for transcriptomic imputation: http://gusevlab.org/projects/fusion/; GTEx v8: https://gtexportal.org/home/datasets/; eQTLgen: https://www.eqtlgen.org/; FinnGen R5 data used for PheWAS analyzes: https://r5.finngen.fi/; Atherosclerosis Risk in Communities (ARIC) study: http://nilanjanchatterjeelab.org/pwas/; deCODE: https://www.decode.com/summa rydata/; Dutch Microbiome Project: https://www.nature.com/articles/s41588-021-00992-y. The datasets supporting the conclusions of this article are included within the article and its additional files (Additional file 1: Table S1 and S2).
