# Supplementary material for: Prioritization of therapeutic targets for cancers using integrative multi-omics analysis
Source: Hum Genomics. 2024 Apr 24;18:42. doi: 10.1186/s40246-024-00571-2 (PMC11040978; doi:10.1186/s40246-024-00571-2)

**Fig. S1. Results of TWASs analysis on cancers in whole blood.** TWAS transcriptome-wide association study.


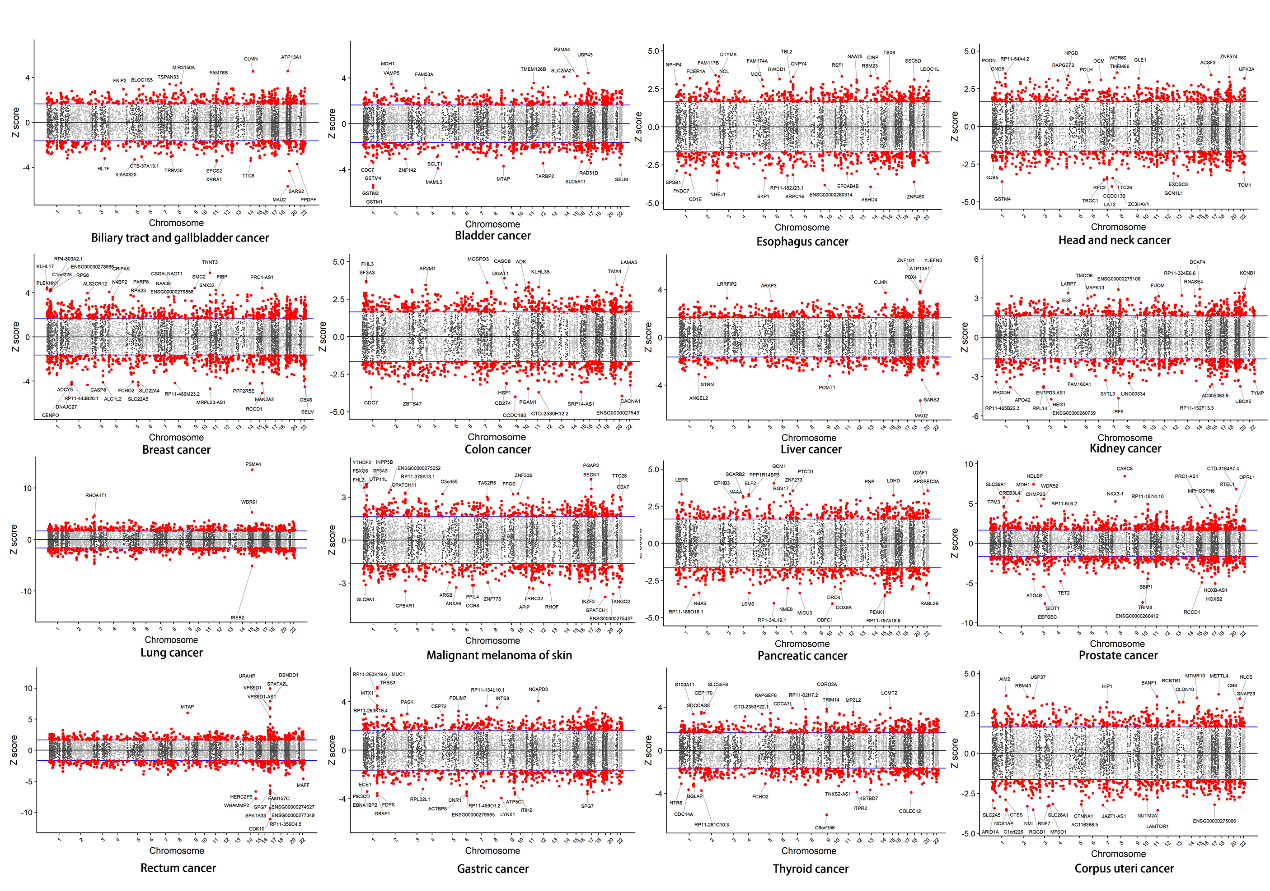


**Fig. S2. Results of TWASs analysis on cancers in specific organ tissue.** TWAS transcriptome-wide association study.


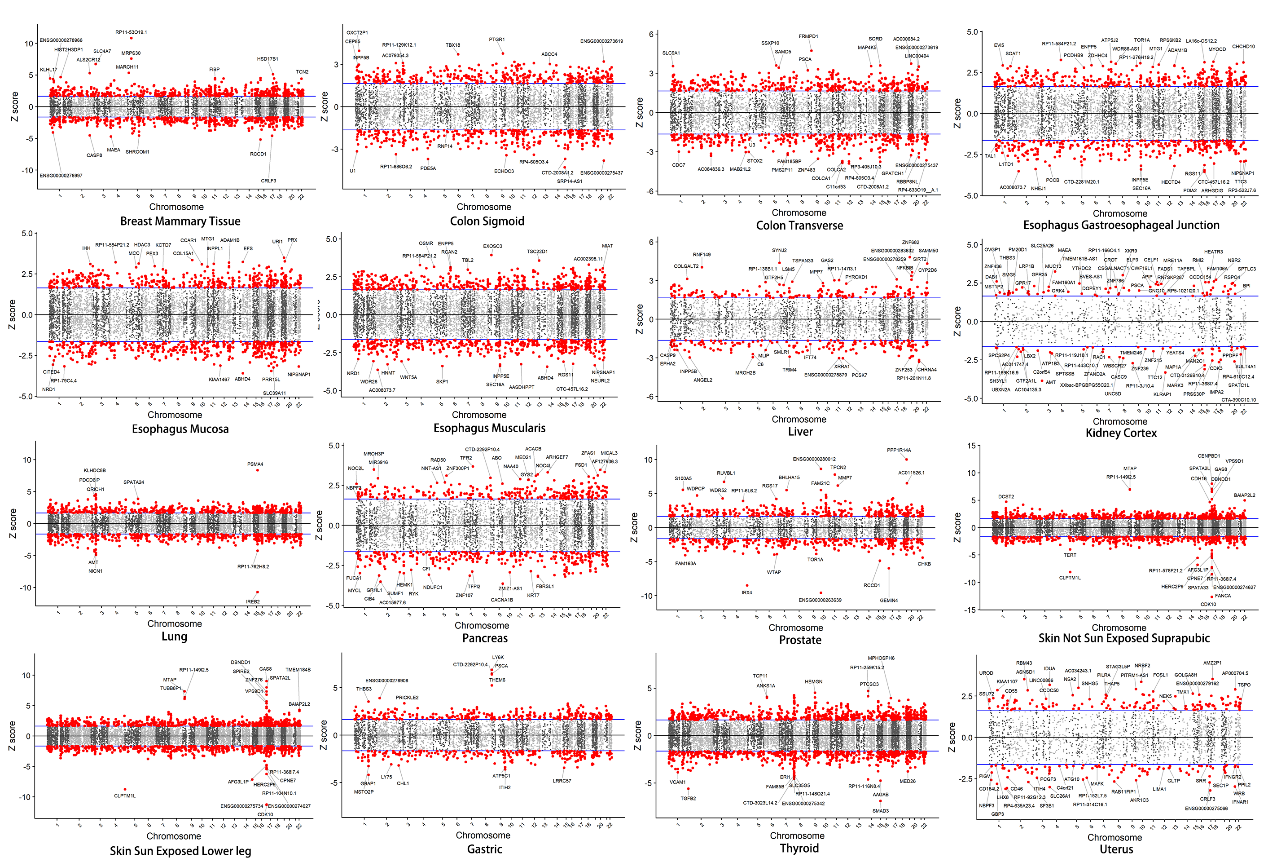


**Fig. S3. Results of PWASs analysis on cancers in whole blood.** PWAS proteome-wide association studies.


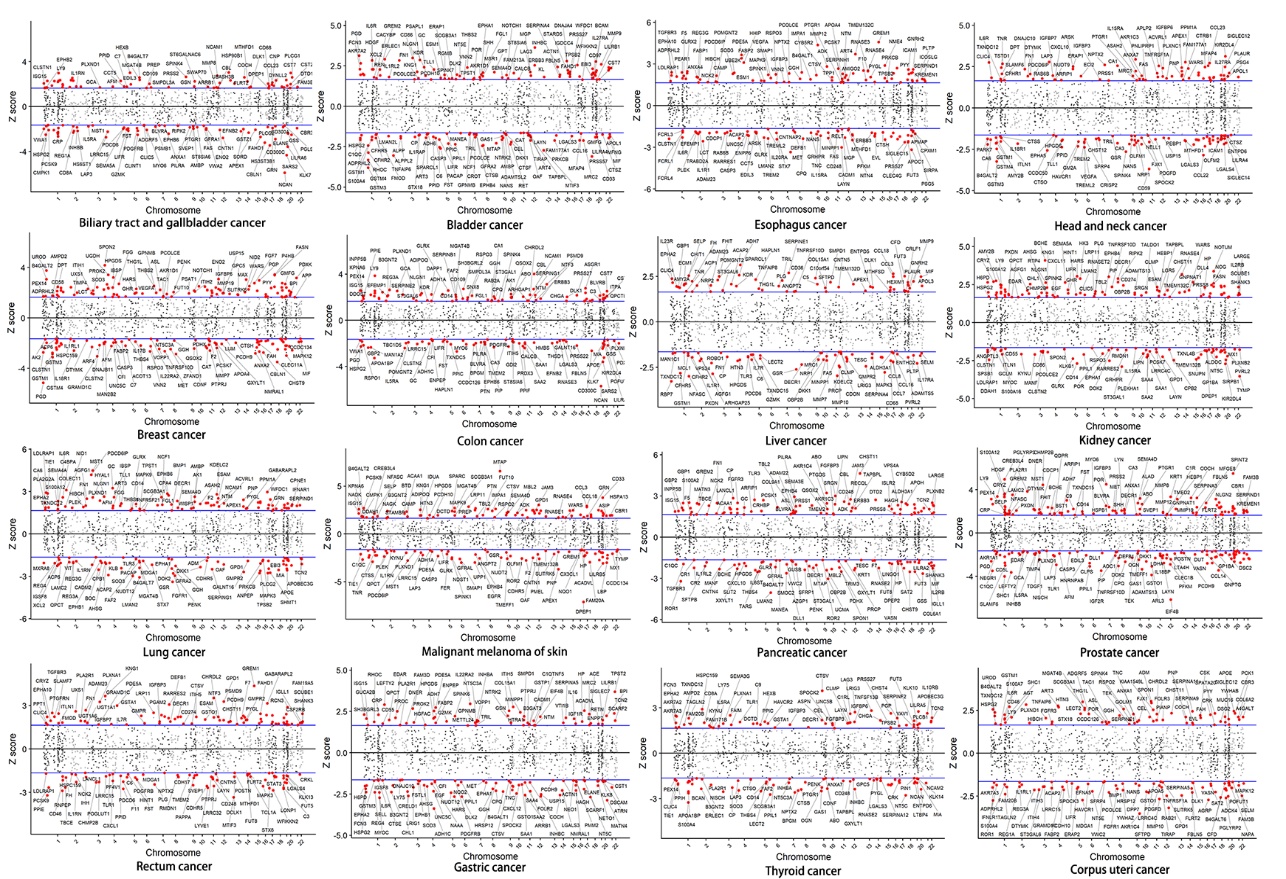


**Fig. S4. Results of differential expression analysis on genes identified in transcriptomic association studies from TCGA and GTEx Database.** TCGA:the Cancer Genome Atlas; GTEx: Genotype-Tissue Expression Project.

**
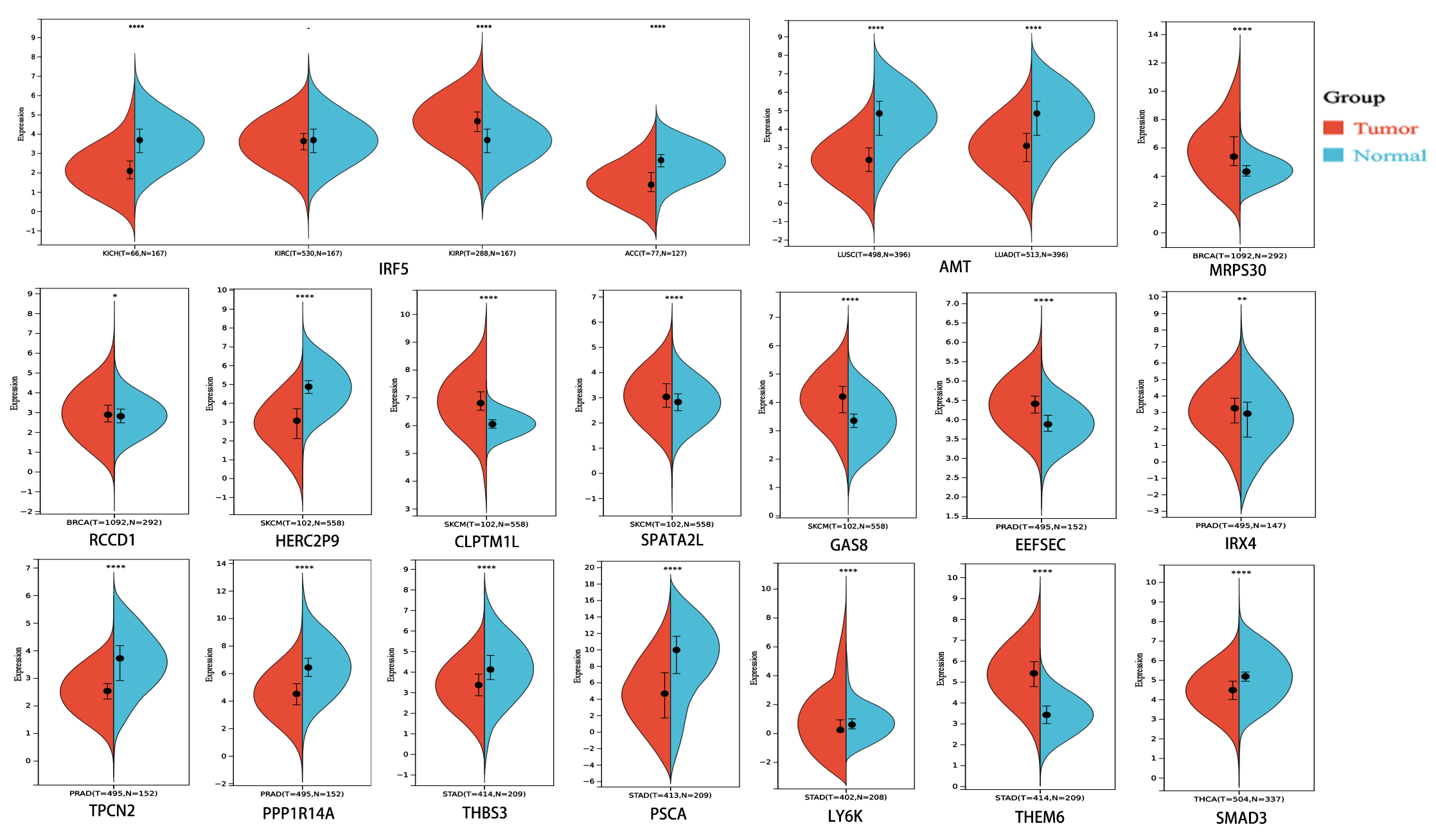
**

**Fig. S5. Results of differential expression analysis on genes identified in proteomic association studies from TCGA and GTEx Database.** TCGA:the Cancer Genome Atlas; GTEx: Genotype-Tissue Expression Project.

**
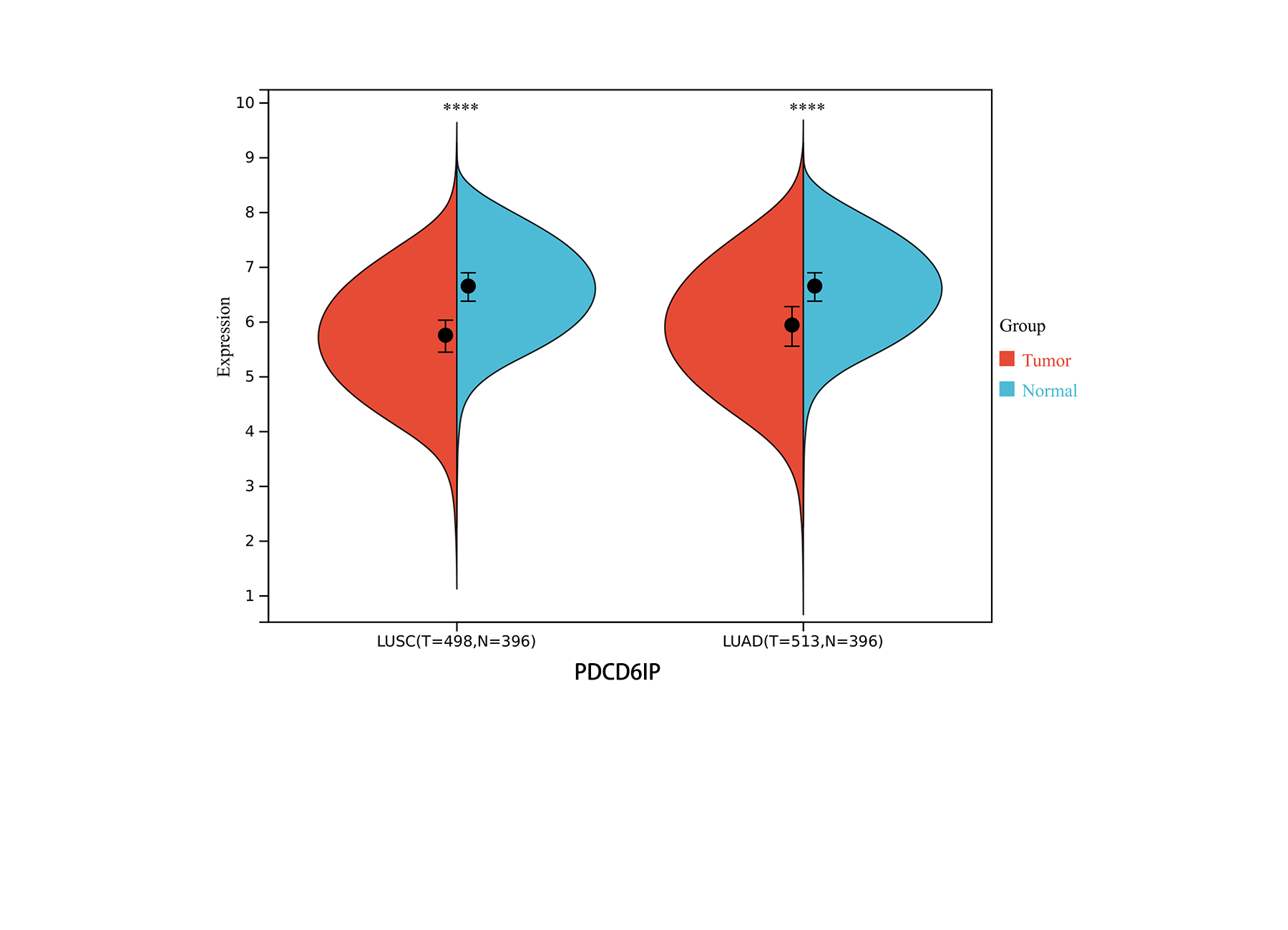
**

**Fig. S6. Results of differential expression analysis on genes identified in druggable genomics association studies from TCGA and GTEx Database.** TCGA:the Cancer Genome Atlas; GTEx: Genotype-Tissue Expression Project.

**
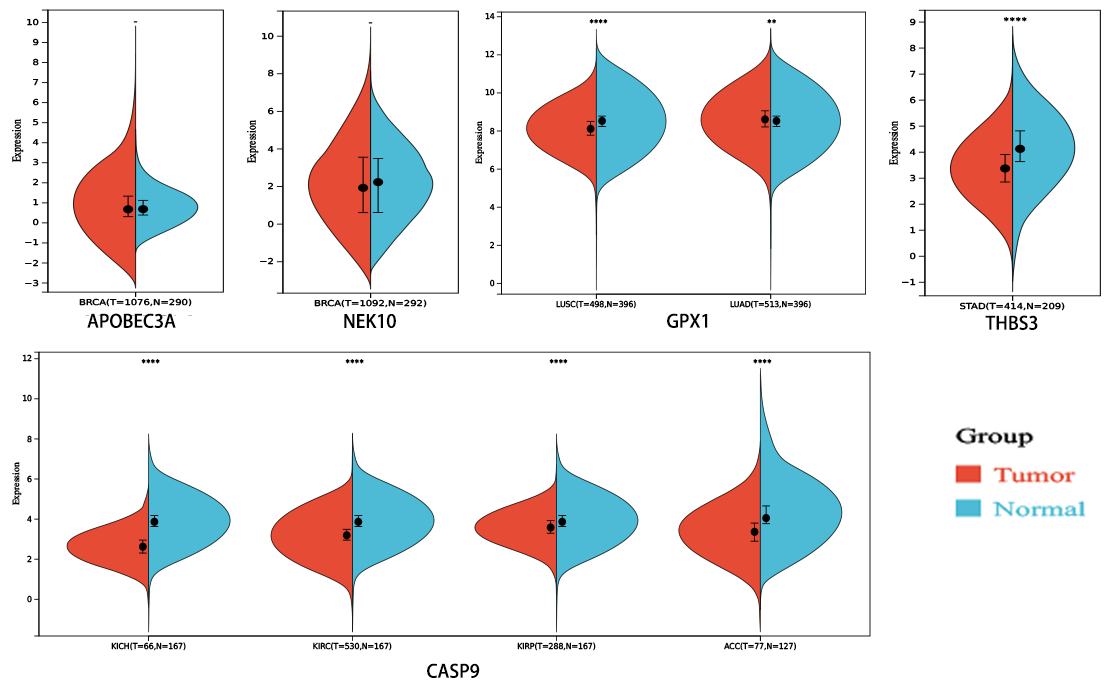
**

**Fig. S7. Results of differential expression analysis on genes identified in druggable genomics association studies from TCGA Database.** TCGA:the Cancer Genome Atlas.


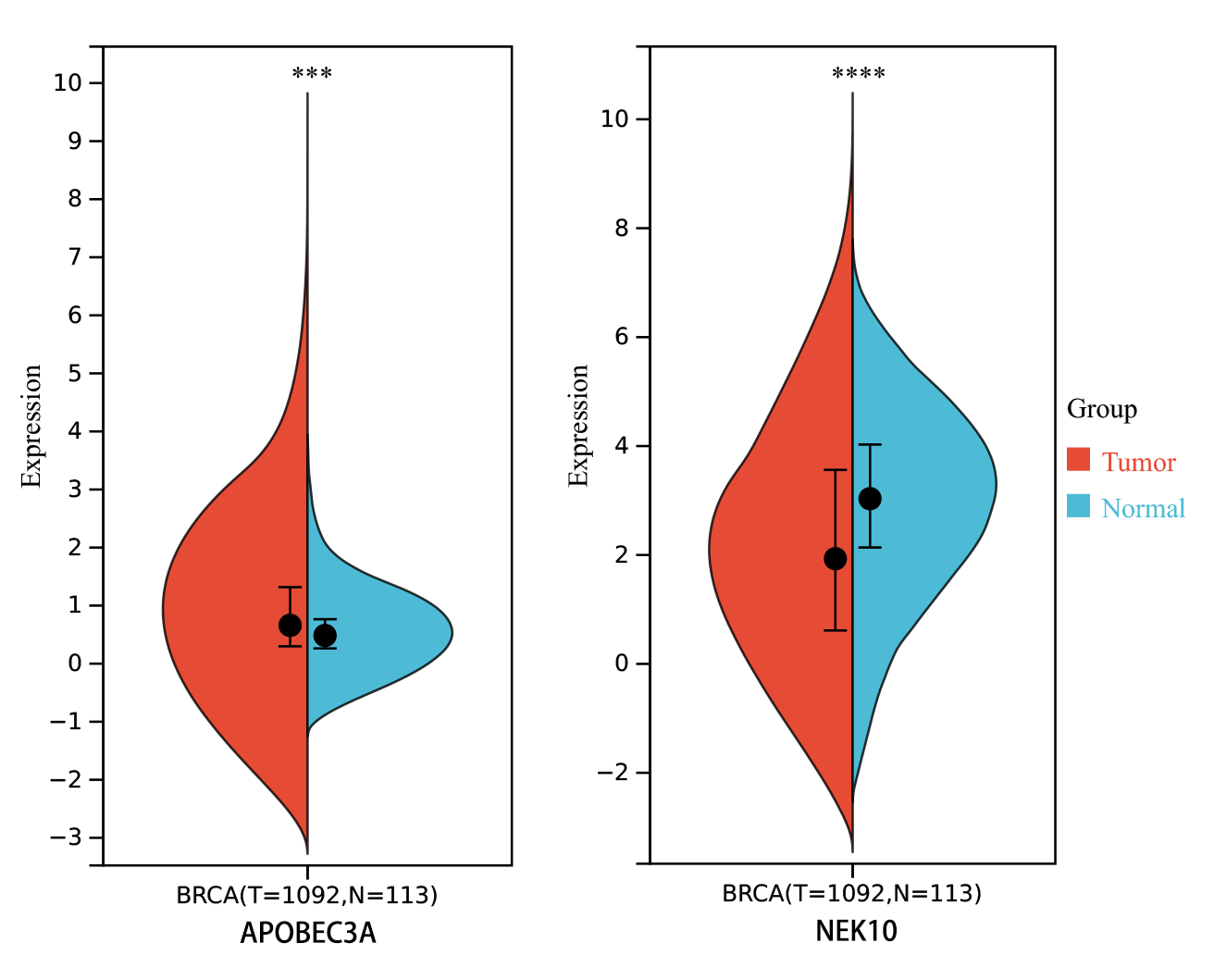


**Fig. S8. Results of enrichment analysis on the genes significantly associated with cancers.** All genes were mainly enriched in NRF2 pathway, regulating of the cellular antioxidant response.


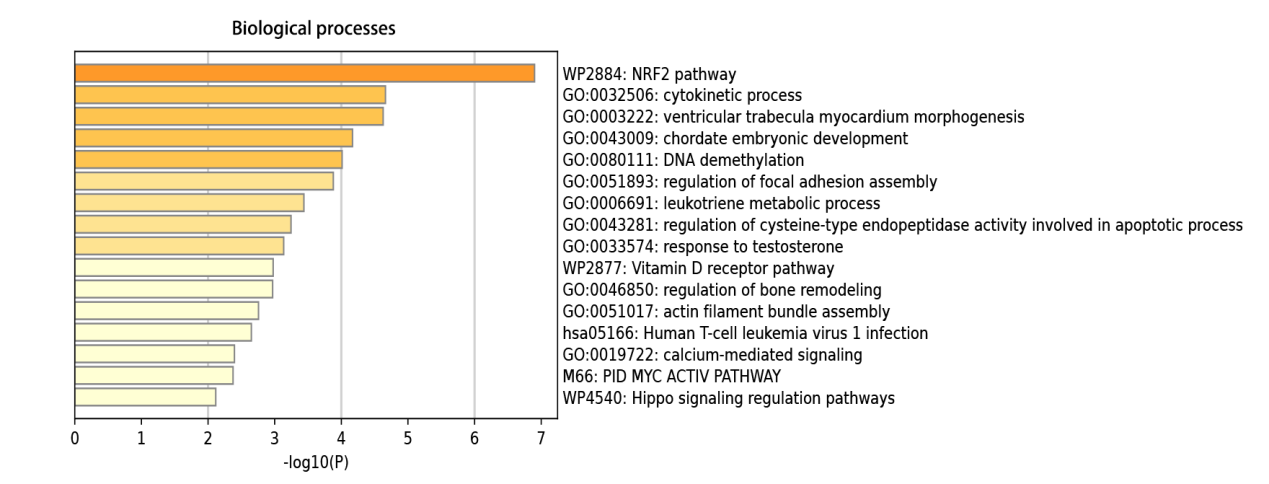

Supplement: Supplementary file 2 — Additional file 2. Fig. S1. Results of TWASs analysis on cancers in whole blood. Fig. S2. Results of TWASs analysis on cancers in specific organ tissue. Fig. S3. Results of PWASs analysis on cancers in whole blood. Fig. S4. Results of differential expression analysis on genes identified in transcriptomic association studies from TCGA and GTEx Database. Fig. S5. Results of differential expression analysis on genes identified in proteomic association studies from TCGA and GTEx Database. Fig. S6. Results of differential expression analysis on genes identified in druggable genomics association studies from TCGA and GTEx Database. Fig. S7. Results of differential expression analysis on genes identified in druggable genomics association studies from TCGA Database. Fig. S8. Results of enrichment analysis on the genes significantly associated with cancers. [file 40246_2024_571_MOESM2_ESM.docx]
